# Challenges in the Analysis of Mass-Throughput Data: A Technical Commentary from the Statistical Machine Learning Perspective

**Published:** 2007-02-16

**Authors:** Constantin F. Aliferis, Alexander Statnikov, Ioannis Tsamardinos

**Affiliations:** 1 Discovery Systems Laboratory, Department of Biomedical Informatics, Vanderbilt University, Nashville, TN, USA.; 2 Department of Cancer Biology, Vanderbilt University, Nashville, TN, USA.

## Abstract

Sound data analysis is critical to the success of modern molecular medicine research that involves collection and interpretation of mass-throughput data. The novel nature and high-dimensionality in such datasets pose a series of nontrivial data analysis problems. This technical commentary discusses the problems of over-fitting, error estimation, curse of dimensionality, causal versus predictive modeling, integration of heterogeneous types of data, and lack of standard protocols for data analysis. We attempt to shed light on the nature and causes of these problems and to outline viable methodological approaches to overcome them.

## 1. Introduction

Recent developments in mass-throughput molecular biology techniques have captured the attention of the medical community in the last few years and promise to revolutionize all aspects of medicine including radically improving medical prevention, diagnosis, development of novel targeted therapeutic agents, personalized treatments, and the ability to predict and monitor the course of the patient. A few characteristic examples of such assaying methods with substantial research applications in the study of cancer prevention, diagnosis and treatment are gene expression microarrays, mass spectrometry, SNP arrays, tissue arrays, array comparative genomic hybridization, and newer variants such as tiled expression arrays, high-resolution mass spectrometry, liquid chromatography-mass spectrometry and others. ([Fortina, 2002], [Valle, 2004], [Khoury, 2003], [Diamandis, 2004], and [Petricoin, 2002]). These methods are capable of producing genotypic, transcriptional, proteomic, and other measurements about cellular function on a massive scale. In the near future such techniques are expected to be combined with novel very-high resolution imaging methods as well as with traditional phenotypic, genetic, and environmental information to create a completely new landscape for biological discovery and improved healthcare.

While the excitement surrounding these developments is growing, researchers have also come to realize that the volume and nature of the data produced pose severe challenges for making biological and medical sense of the data and using it optimally. Sound and comprehensive data analysis is critical to the success of modern molecular medicine research that involves collection and interpretation of mass-throughput assays. For example, the high dimensionality of the data (indicative examples are 10,000, 50,000 and 500,000 gene and SNP microarrays, 10,000 and 300,000 m/z value mass spectrometry data, and >5,000,000 variable liquid chromatography-mass spectrometry data) combined with limited samples has challenged traditional multivariate analysis methods, has often incurred large numbers of false positives for statistical tests, and has brought into sharp focus the need for multidisciplinary teams to address aspects of the data analysis (eg, engineers and applied mathematicians to process signals and extract features from arrays and mass spectrometry data, machine learning and pattern recognition experts to derive complex multivariate “signatures”, and biostatisticians to conduct power-size analyses or to design appropriate data collection study designs).

In this technical commentary, we review and discuss some recurring problems in the analysis of such data. We believe that it is necessary that not only analysts but also all members of research teams and other parties involved with such studies (eg, biologists and physicians, members of funding and regulatory agencies) should possess at least a fundamental understanding of the major data analytic issues. These challenges (that are independent of the specific mass-throughput platform) include: over-fitting, that is the phenomenon whereby an apparently good model fails to generalize well in future samples; estimating the generalization error for predictive models; the “curse of dimensionality”, ie, the set of data analysis problems deriving from large numbers of predictors especially when combined with small samples; and building causal in addition to predictive models. Other challenges relate to the integration of heterogeneous types of data and addressing the lack of standard protocols and the proliferation of alternatives for data analysis, especially in high dimensions. We attempt to shed light on the nature and causes of these problems and to outline viable conceptual and methodological approaches to overcome them. To make the paper as simple as possible, we illustrate these problems by simplified examples that hold independently of the context of application. We further provide references to published studies that apply these principles in real-world cancer as well as other biological data.

Clearly some other technical, organizational, ethical and science culture-related issues exist, that although important, they are not addressed in the present paper. Such issues, for example, involve challenges that deal with specific platform assay validity and reproducibility, the study of how to optimally deliver the results of the respective literature to physicians at the bedside, regulating molecular medicine modalities for safety, explaining complex models to practitioners, exploring proper ways to build and maintain interdisciplinary teams, storing, protecting, and retrieving patient data, etc. We choose to focus on only the platform-independent data analysis challenges in the present paper, not only because of the enormity of the combined problem landscape which would render any single paper addressing the totality of all the mentioned problems shallow and operationally weak, but also because, conceptually, methodological data analysis challenges cut across the spectrum of molecular medicine research, and they can be addressed in a coherent manner fairly independently from the remaining challenges. For examples of reviews of platform-specific data analysis challenges, we refer the reader to [Listgarten, 2005] and [Simon, 2003].

In the present paper we adopt a statistical machine learning perspective, not only because a great deal of work in the literature so far has been accomplished using such techniques^1^, but primarily because as we show in the paper this perspective is well-suited to illuminate the issues surrounding mass-throughput data analysis.

In section 2 we provide a brief introduction to statistical machine learning, while in Appendix I a complementary glossary explains all major technical terms and abbreviations used in the paper but not defined explicitly in the text. Section 3 contains the substantive part of the paper (problems, their causes and possible ways to address them), while section 4 offers concluding remarks.

## 2. Statistical Machine Learning: A Brief Introduction

Machine Learning is the broad field of computer science that studies the theory and practice of algorithms and systems that learn. A learning algorithm or system is one that improves performance with experience. Such algorithms are called “learners”. Learners may learn symbolic (eg, first-order logic) or non-symbolic models of data. The latter are studied by *Statistical Machine Learning* (as opposed to *general* machine learning that deals with symbolic learning as well)^2^. In genomics and molecular medicine for example, a statistical machine learning diagnostic system may improve its ability to diagnose patients with experience provided and encoded as the results of micro-array profiles (or profiles from another assaying technology) of past patients. Examples of well-established learning methods with applications in biomedicine are: Artificial Neural Networks, Bayesian Networks, restricted Bayesian Classifiers (eg, simple Bayesian Classifier, Tree-Augmented Networks), Decision Tree Induction, Genetic Algorithms, Clustering methods, Rule Induction, Ensemble Classification (eg, Voting, Bagging, Boosting, Bayesian Model Averaging), Support Vector Machines, Hidden Markov Models, Causal Inference methods, and many others (see [Mitchell, 1997], [Hastie, 2001], [Duda, 2001], and [Weiss, 1991]).

**Figure 1.**
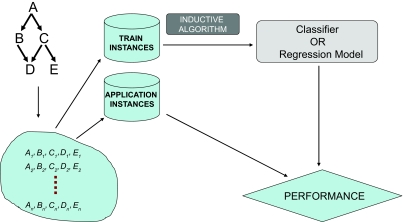
Diagrammatic representation of supervised learning. A biological or experimental causal process generates data. From this population of data a dataset of training instances is sampled randomly. An inductive algorithm learns a classification or regression model from the training data. The model is applied (or tested) in independently sampled application (or test) data.

An important concept in Statistical Machine Learning is the distinction between *supervised and unsupervised learning.* In supervised learning we give to the learning algorithm a set of instances of input-output pairs; the algorithm learns to predict the correct output that corresponds to some inputs (not only previously seen ones but also previously unseen ones (we call this “generalization”)). For example, a researcher/analyst may present to a learning algorithm array gene expression measurements from several cancer patient cases as well as normal subjects; then the learning algorithm induces a classifier that can classify a previously unseen subject to the correct diagnostic category given the gene expression values observed in that subject. Figure 1 outlines supervised learning diagrammatically. An important thing to notice is that the learning algorithm induces from data a model (classifier or regression model) that is capable of predicting discrete classes for unlabeled patients (classification) or of predicting the unknown continuous values of a continuous response variable of interest (regression). The model is not the learner but the output of the learner.

**Figure 2.**
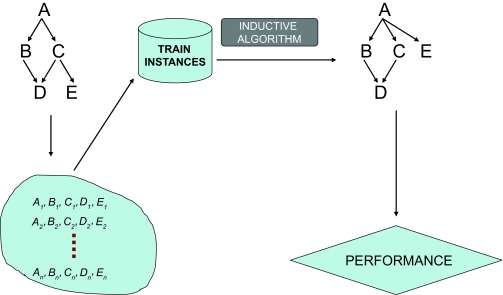
Diagrammatic representation of unsupervised learning. A biological or experimental causal process generates data. From this population of data a dataset of training instances is sampled randomly. An inductive algorithm learns a structural model from the training data. The model is verified by experiments, comparison to known biological knowledge, or other means.

In unsupervised learning, the researcher/analyst typically seeks to discover categories or other structural properties of the domain. For example, a researcher may give to a learning algorithm gene expression measurements of patients with cancer as well as normal subjects; the algorithm then finds sub-types (“molecular profiles”) of patients that are very similar to each other, and different to the rest of the sub-types (eg, see [Bhattacharya, 2003]). Or another algorithm may discover how various genes interact with each other to affect development of cancer. Figure 2 outlines unsupervised learning diagrammatically.

The branch of Machine Learning that deals with the theoretical properties and feasibility of learning under various conditions is called Computational Learning Theory (COLT). Sometimes Machine Learning explores questions that fall outside the traditional scope of classical Statistics, for example learning with continuous feedback from the environment in autonomous robotic agents (reinforcement learning), learning from relational databases (relational learning), or learning interesting patterns with no pre-specified outcome of interest (clustering, and more generally, concept formation and unsupervised learning). Conversely, sometimes Statistics deals with questions that fall outside the traditional scope of Machine Learning, for example constructing specialized research designs that minimize sample or maximize statistical power, or estimating the confidence limits of quantities of interest in specific sampling contexts. Often both Statistics and Machine Learning solve the same type of problem using different approaches, for example deriving classification models using a generative model approach (the statistical approach, as embodied in logistic regression), or finding a highly predictive decision surface (the machine learning approach, as embodied in Support Vector Machines). In addition, numerous Machine Learning methods build upon statistical theory and techniques. Thus Machine Learning and Statistical approaches are quite often synergistic for attacking hard modeling and discovery data analysis problems. An accessible and extensive on-line tutorial on Machine Learning methods and biomedical case studies is available from http://www.dsl-lab.org. Some excellent introductory texts are [Mitchell, 1997], [Hastie, 2001], [Duda, 2001], and [Weiss, 1991].

## 3. Methodological Challenges & Solutions

### 3.1. Over-fitting & estimation of a model’s generalization error

#### (a) What is Over-fitting?

**Figure 3.**
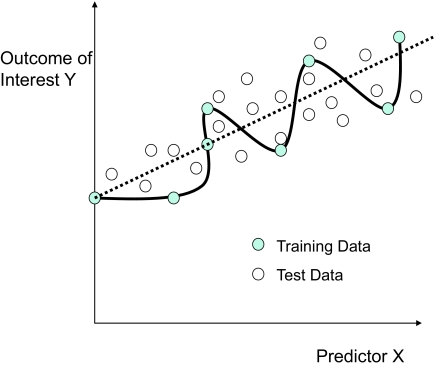
An example of over-fitting. The solid line represents a (non-linear) regression model of *Y* given predictor variable *X*, where the training data are represented by the solid circles. This model perfectly fits (“predicts”) the training data (ie, the training error is zero). The true relationship however is linear with Gaussian noise (shown by a dashed line). Prediction error on future data (shown with white circles) is likely to be both higher than zero and higher than the prediction by the optimal linear model.

*With the term “over-fitting” we denote the phenomenon in which a classification or a regression model is exhibiting small prediction error in the training data but much larger generalization error in unseen future data*. Intuitively, a learning method over-fits when it does not learn the general characteristics of the function that predicts the data but the non-reproducible peculiarities of the specific sample available for analysis. An example is shown in Figure 3 where a regression model for *Y* is build from predictor variable *X*. The true relationship is linear with Gaussian noise and is shown with the dashed line while the solid line represents a regression model learned from the training data shown with solid circles. The training error is zero because the model fits exactly (“predicts”) each training case but the true generalization error (ie, the error in the general population where the data came from) is much higher. As an example, we show some likely future data denoted by the white circles. The generalization error of the over-fitted model is also higher than the one achieved by the optimal linear model. Obviously, in any non-trivial data analysis we can never exactly estimate with absolute certainty the true generalization error of a model. However, modeling principles exist that one should follow to reduce the likelihood of over-fitting. Moreover, methods also exist that provide bounds and confidence intervals on the estimation error allowing detection of over-fitting. Both of these methods are explained in detail in Section 3.2.

The training error is typically a misleading estimate of the generalization error, unless treated appropriately as we will discuss in more detail later in the present section. In mass-throughput data, such as microarray gene expression data for example, we are dealing with thousands of variables (eg, gene probes) used as continuous predictors and a much smaller sample (typically at the order of a few hundred patients in the best case). In such situations each specific sample has a unique combination of predictor values that can be used to identify that sample (and its associated known phenotype or outcome). Hence even the weakest of learning methods, a lookup table (also known as “rote learner”) achieves 100% classification accuracy in the training data. Future applications of that learner to other independently and identically distributed samples would be uninformative (ie, no better than chance) however.

Unless special precautions are taken, over-fitting is easier to occur when using learning methods capable of approximating very complex functions, and when the data has a large number of predictors and small sample size. Some authors define loosely over-fitting as synonymous or equivalent to having too many free parameters, or “over-specifying” the model space by having many more predictors than samples (another term for this phenomenon is “overparameterization”). However these factors are *merely facilitating factors*, and strictly speaking they are neither necessary nor sufficient for over-fitting, as we will show below.

#### (b) What causes over-fitting?

##### The Bias-Variance Error Decomposition Analysis View

One way to study the problem of over-fitting is by the bias-variance decomposition analysis. According to this analysis, for any predictive model produced by a classifier given a specific dataset, the (expected) generalization error can be decomposed into three components: the *bias,* the *variance*, and the *noise.* Let us denote by *O* the optimal prediction model that can be obtained for this task (over all possible learners), *L* the optimal model for the problem that can be generated by *a specific learner* (ie, this model among the models that can be produced by the specific learner exhibits the least error over the whole sampling population), and *A* the actual model the classifier has learned with the given dataset.

The noise component corresponds to the inherent uncertainty of the relationship we try to learn from data, ie, the error of the optimal model *O*, and so it is irreducible by definition. When the target is a deterministic function of the data the noise component of the error is zero, and it is non-zero otherwise. The bias component corresponds to the difference of *O* and *L*. For example, if a learner produces linear classifier models only, the bias component is the expected error of the difference of the optimal linear model relative to the overall optimal prediction model (that may be non-linear for example). The bias is zero for a learner that is capable of learning an optimal model for the learning task. The variance component is the difference of *L* and *A*. The variance component is a reflection of randomness in the sample available for training, is independent of the true value of the predicted example, and zero for a learner that always makes the same prediction independent of the training dataset. In summary, the error of a model is decomposed to the error of the learnt model relative to the optimal model the specific classifier is capable of producing (variance) (*L-A*), the error of the latter model relative to the optimal model (bias) (*O-L*), and the error of the optimal model *O* (see [Domingos, 2000] and [Friedman, 1997] for mathematical details and examples of analyses of specific learners).

**Figure 4.**
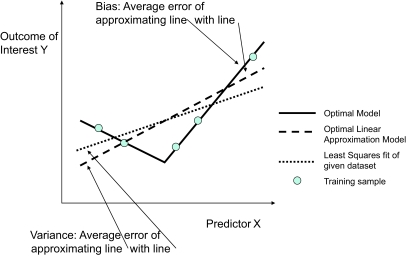
An example of bias-variance decomposition. The true relationship (optimal model) between the predictor X and outcome Y is shown with the bold line. It is deterministic so there is no noise component. The optimal linear least-squares fit is shown with the dashed line. The bias component for this task is the least-squares difference between these two models (averaged out on all values of X). The linear least-squares fit given a specific training dataset (shown with the circles) is denoted by the dotted line. The variance component for this task and dataset is the difference (averaged out on all values of X) between the dotted and the dashed lines.

An example is shown in Figure 4. The true relationship (optimal model) between the predictor *X* and the outcome *Y* is shown with the bold line. It is deterministic, so there is no noise component. The error is measured by mean squared difference. The optimal linear least-squares fit is shown with the dashed line. The bias component for this task is the mean least-squares difference between these two models. The linear least-squares fit given a specific training dataset (shown with the circles) is denoted by the dotted line. The variance component for this task and dataset is the mean squared-difference between the dotted and the dashed lines.

The bias-variance decomposition helps us understand the conditions under which a classifier is likely to over-fit. Notice that the bias is a function of the classifier and the actual classification task, while the variance is a function of the classifier and the given dataset. A classifier with high-bias (eg, Simple Bayes) is not able to learn as complicated functions as one with low-bias (eg, *k*-Nearest Neighbors and the rote-learner described above). However, when learning from a small number of training examples, the high-bias classifier may often out-perform the low-bias one because the latter may have a much larger variance component. This observation is particularly important in analyzing mass-throughput data as currently such datasets have relatively few training cases. It readily explains, for example, the success of high-bias (eg, linear) classifiers in such datasets. The bias-variance decomposition may also explain why ensemble methods work well: while they increase the complexity of the learned decision surface relatively to a single classifier, ensembling typically reduces the variance component of the error. In addition, the decomposition partially explains why modern methods such as Support Vector Machines are as successful across many datasets and tasks: these methods control the bias-variance trade-off relative to the available sample by not allowing the decision surface complexity to grow out of proportion to the available sample (more on Support Vector Machines and this issue in the sections below). In contrast, the bias of the Simple Bayes classifier is independent of the sample size.

#### (c) What causes over-fitting?

##### The Computational Learning Theory perspective

A second perspective on over-fitting is provided by Computational Learning Theory (COLT), which formally studies under which conditions learning is feasible and provides several bounds for the generalization error depending on the classifier used, the definition of error to be minimized (eg, number of misclassifications), and other assumptions. While theoretical results in classical statistics typically make distributional assumptions about the data (ie, the probability distribution of the data belongs to a certain class of distributions), COLT results typically make assumptions only about the class of discriminative model considered. Notice though, that it may be the case that an optimal discriminative model never converges to the probability distribution of the data.

COLT research has defined several mathematical models of learning. These are formalisms for studying the convergence of the errors of a learning method. The most widely-used formalisms are the *VC (Vapnik-Chervonenkis) and the PAC (probabilistically approximately correct) analysis.* A VC or PAC analysis provides bounds on the error given a specific classifier, the size of the training set, the error on the training set, and a set of assumptions, eg, in the case of PAC, that an optimal model is learnable by that classifier. Typical PAC bounds, for example, dictate that for a specific context (classifier, training error, etc.) the error will be larger than epsilon with probability less than delta, for some given epsilon or delta. Unlike bias-variance decomposition, COLT bounds are independent of the learning task. From the large field of COLT we suggest [Anthony, 1992] and [Kearns, 1994] and the recent tutorial by [Langford, 2005] as accessible introductions.

It is worth noting one interesting theoretical concept from COLT that is pertinent to over-fitting. The VC (Vapnik-Chervonenkis) dimension (not to be confused with the VC model of learning above) is (informally) defined as the maximum number of training examples that can be correctly classified by a learner for any possible assignment of class labels. The VC dimension of the classifier is a quantity that frequently appears in estimation bounds in a way that all else being constant, higher VC dimension leads to increased generalization error. Intuitively, a high-bias classifier has low VC dimension and vice-versa. An example of VC bound follows: if VC dimension *h* is smaller than *l*, then with probability of at least 1-*η*, the generalization error of a learner will be bounded by the sum of its empirical error and a confidence term defined as

$$\sqrt{\frac{h\left(\text{log}\frac{2l}{h}+1\right)-\text{log}(\eta /4)}{l}}$$

(notice that this bound is independent of dimensionality of the problem; this is an important observation that we revisit when we discuss how to overcome the curse of dimensionality) [Schölkopf, 1999].

The number of parameters of a classifier *does not necessarily correspond to its VC dimension.* In [Herbrich, 2002] (Chapter 4) examples are given of a classifier with a single parameter that has infinite VC dimension and classifiers with an unbounded number of parameters but with VC dimension of 1. Thus, a classifier with a large number of parameters (but a low VC dimension) can still have low error estimates and provide guarantees of non-over-fitting. In addition, some of these bounds are non-trivial (ie, less than 1) even when the number of dimensions is much higher than the number of training cases. This proves unequivocally that *learning is possible in the situation common in mass-throughput data where the number of observed variables is much higher than the number of available training sample.* Many popular classical statistical predictive modeling methods in contrast break down in such situations. The mentioned COLT results also justify our prior assertion that *over-fitting is not equivalent to a high number of parameters.* Finally, certain heuristics used in practice, such as the Occam’s razor which can be stated as “everything else being equal, choose the simplest explanation (model) that fits the data”, are naturally explained using computational learning theory since the simplest models provide the smallest error bounds [Langford, 2005].

Unfortunately, many of the estimation bounds provided by COLT are not tight for the number of samples available in mass-throughput data analysis, but current research is promising in deriving bounds that are more relevant in practice. In addition, COLT results often drive the design of classifiers with interesting theoretical properties, robust to the curse of dimensionality, and empirically proven successful, such as Support Vector Machines (discussed in detail later).

#### (d) What causes over-fitting?

##### Multiple Validation

Our discussion so far has dealt with estimation of the error of a single predictive model. Another major source of over-fitting, not encapsulated by the single-learning setting of the theoretical frameworks mentioned above is *multiple validation3*. In this situation the researcher builds a series of models using a fixed training sample and estimates the error of each one of them using either a fixed test dataset or using the training error and theoretical bounds. Then the researcher chooses the model that has the lowest estimated error. Unfortunately, the variance of the generalization error estimates by the empirical error in a small-sample validation set is high, thus the model with the lowest validation error may not be the model with the lowest (true) generalization error ([Braga-Neto, 2004], [Weiss, 1991]).

Over-fitting by multiple validation can occur both by means of computationally intensive data fitting schemes (eg, genetic algorithm search with no special precautions against over-fitting) but also by repeated manual analysis of the data (by the same or other research groups even, as happens for example in data analysis competitions and repeatedly analyzed datasets in the public domain) in which the data is analyzed by many different approaches (or different parameter settings of the same approach) until a model with low error in the training or validation data is identified^4^.

**Figure 5.**
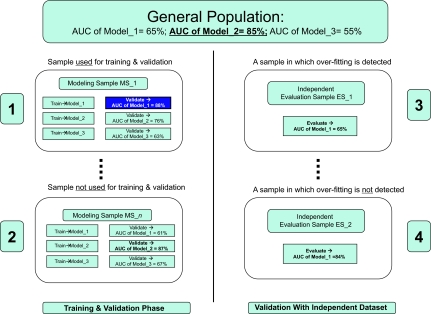
Independent sample validation does not always detect over-fitting (see text for details).

In Figure 5 we examine some typical over-fitting scenarios via multiple validation. Assume the very common situation of a researcher who is interested in developing a model that is highly discriminatory for cancer (or some other response variable of interest, such as clinical outcome, metastasis, response to treatment etc.). Without loss of generality, and for clarity of presentation let us assume that only 3 models can be built to fit the data and that in the general population, model_2 is the best and has a true (ie, population-wide) area under the ROC curve (AUC) of 0.85. The researcher has access to one sample set sampled from the general population uniformly randomly. For any population of non-trivial size *n* and given enough predictor variables there is a very large number of non-equivalent samples of size *k* such that *k is much smaller than n.* Assume that the one sample the researcher sees is “1” on the left top corner (all other samples like “2” are not available to the researcher). The researcher fits all three possible models and estimates their error in the general population by some variant of cross-validation (see section 3.2(a)). If the sample size is small then all these procedures will produce an error estimate that varies a lot from sample to sample. In our example the estimate for model_1 is 88% instead of the true 65%, the estimate for model_2 is 76% instead of 85%, and for model_3 is 63% instead of 55%. On the basis of these estimates the researcher chooses model_1 as the best model and expects that model_1 will exhibit AUC of 88% in future applications. Since however the true AUC for this model is only 65%, the model is over-fitted, that is, it matches well the sample but not the population.

**Figure 6.**
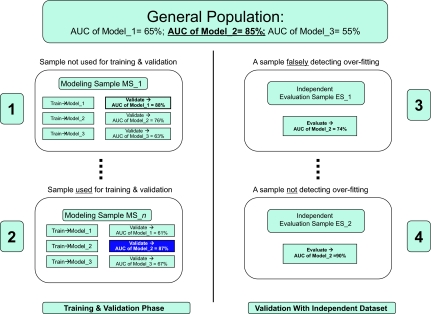
Independent sample validation may falsely conclude over-fitting (see text for details).

Now let us assume that our researcher is aware of this possibility and decides to accept the model only if in addition to good cross-validation error the model also exhibits good performance in a completely independent sample. Unfortunately as long as the independent sample is small, the same problems regarding high variance of the error estimate may well affect the researcher’s conclusions. Specifically if out of the many possible independent test samples (depicting to the right hand-side of Figure 5) the independent sample seen by the researcher is “3”, model_1 has an AUC closer to the true (ie, population) one, and the over-fitting *will* be detected. However if the researcher is unlucky enough to see dataset “4” as the independent data (but not “3” or any other), she will not detect over-fitting since in test sample “4” model_1 exhibits higher AUC than the true one. The researcher will confidently publish model_1 with the claim of no over-fitting. These examples show that although some believe that a completely independent validation with low-error precludes over-fitting this is *not* always the case. Furthermore, if the researcher has access to dataset “3” over-fitting will be *established but not prevented.* The researcher will face then the following set of hard decisions: (a) publish a negative finding; or (b) repeat training and try to develop/evaluate a new set of models (in our example we assumed three possible models only but typically there is an infinity of them); or (c) return to training and use different modeling methods; or (d) abandon the hypothesis without trying to publish. Options (b), and (c) are prone to over-fitting the (formerly but no longer) “independent” dataset in a subsequent round of model building and validation since, assuming enough modeling flexibility, eventually some model that is good for both the training and the (formerly but no longer) “independent” datasets will be found. Option (d) does not disclose potentially valuable information (eg, other researchers may attempt to test the same hypothesis/model building but the current experiment at a minimum suggests a way *not* to do the analysis and is thus of use as long as subsequent analyses are not done on the same data (to avoid over-fitting by other groups as in cases (b), (c) above). We return our attention to option (a) and show (Figure 6) that, unfortunately, a negative finding is not unequivocally supported by this experiment either.

In this second example scenario set, the researcher has access to sample set “2” (instead of “1”) for training and validation and to either sample set “3” or “4” for independent validation. In this example the estimate for model_1 is 61% instead of the true 65%, the estimate for model_2 is 87% instead of 85%, and for model_3 is 67% instead of 55%. On the basis of these estimates the researcher chooses model_2 as the best model and expects that model_2 will exhibit AUC of 87% in future applications. Since the true AUC is 85%, the modeling is minimally over-fitted, and thus it matches well the sample and the population. Notice however that an independent validation of model_2 in independent sample “3” gives AUC of only 74%, while in independent sample “4” AUC is 90%. If the researcher has access to “3”, she will conclude that model_2 is over-fitted, while if she has access to “4” she will conclude that the model is not over-fitted. This example shows that indication of over-fitting via independent sample validation is only an indication, not proof for over-fitting. *The two sets of scenarios taken together show that validation by independent prospective testing is neither sufficient nor necessary for either preventing or detecting over-fitting when training and/or testing sample size is small.*

### 3.2. Methods to prevent or detect over-fitting and to estimate error accurately

The following have been used with varying degrees of success in biomedicine and other fields to detect and prevent over-fitting and to estimate error:

#### (a) Cross-validation & bootstrap schemes

In cross validation, the available data is split and a part of the data is used to fit a model while another part is used to estimate generalization error. There are at least three maj or variants of cross-validation: (1) the *holdout estimator* (single training/testing split method), (2) the *N-fold cross-validation* (divides the data in N non-overlapping subsets such that their union is the full data and uses each subset for testing (testing set) and its complement for training (training set); the reported error is the average error over N testing sets), (3) the *leave-one-out cross-validation* procedure (same as N-fold cross-validation with N = sample size) [Weiss, 1991]. The above three methods have different characteristics depending on the data distribution (eg, see [Kohavi, 1995], [Azuaje, 2003], and [Braga-Neto, 2004]). Usually, when the sample size is small, the cross-validation methods may suffer from high variance making the performance estimates unreliable. Recent work [Braga-Neto, 2004] proposes to repeat N-fold cross-validation multiple times over different cross-validation splits of the data and report the average performance over multiple runs of N-fold cross-validation. This strategy allows to reduce the split variance of the data which is due to different cross-validation splits (some of which may accidentally be “good” while others may be “bad” in terms of classification performance).

**Figure 7.**
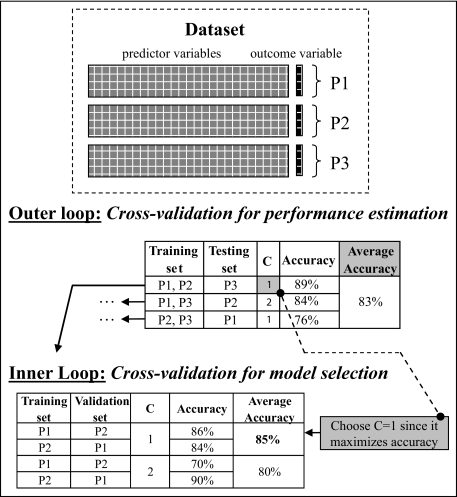
Pictorial simplified example of *nested 3-fold cross-validation.* The data are split into 3 mutually exclusive sets of samples: P1, P2 and P3. The performance is estimated in the outer loop by training on all sample sets but one, using the remaining one for testing. The average performance over testing sets is reported. The inner loop is used to determine the optimal value of the classifier’s parameter C that takes values “1” and “2” (in a cross-validated fashion). This value of parameter C is used for training in the outer loop. A detail algorithmic description is provided in [Statnikov, 2005b].

It is also worthwhile to mention *nested N-fold cross-validation* as a variant of *N-fold cross-validation* that encapsulates one layer of cross-validation inside another one. The inner layer is used to try out different parameters (corresponding to models) and choose ones that work best for the given distribution; the outer layer is used to evaluate the best parameters found in the inner layer. See Figure 7 for an example. This method is quite powerful for detecting over-fitting and estimating the generalization error conservatively. It works very well in microarray and other high-dimensionality data (see [Statnikov, 2005b], and [Aliferis, 2003c]). However in very small samples it is subject to the same high-variance issues that regular cross-validation faces.

Contrary to cross-validation, bootstrap estimators require sampling with replacement of a large number of bootstrap samples; several methods exist to estimate the generalization error thereafter (see [Efron, 1983], [Efron, 1994], and [Efron, 1997]). While bootstrap methods have lower variance of the predictions, their computational cost is typically high and they often suffer from increased bias^5^ (see [Braga-Neto, 2004], and [Efron, 1997]).

Finally, we would like to note that any form of cross-validation or bootstrap estimation does not prevent over-fitting, but detects it. However, proper application of cross-validation procedures prevents *publication* of over-fitted models.

#### (b) Theoretical bounds on error

Analysis of the type of classifier employed, or distributional assumptions about the data can be used to provide bounds of the generalization error based on the training error. Such bounds are provided by Statistical Theory for statistical predictive models and by Computational Learning Theory for machine learning predictive models ([Vapnik, 1998], [Anthony, 1992], [Kearns, 1994], and [Herbrich, 2002]). Typically, statistical methods attempt to estimate the probability distribution of the data. The distribution estimate can then be used to create a predictive model. Theoretical results from statistics can then sometimes provide bounds on how good is the approximation of the true distribution by the learned one based on the training sample and a bound on the generalization error. However, there are cases where knowledge of the data distribution is not necessary for optimal classification or regression. For example, when the task is to minimize the *number* of mistakes a classification model makes on discriminating cancerous tissue from normals given gene expression data, there is no need to predict the exact probability of the tissue being cancerous: any such probability higher than 50% leads to the same optimal classification. This “common sense” idea is captured in the following quote by Vapnik: “When solving a given problem one should avoid solving a more general problem as an intermediate step” [Vapnik, 1998].

#### (c) Feature selection

**Figure 8.**
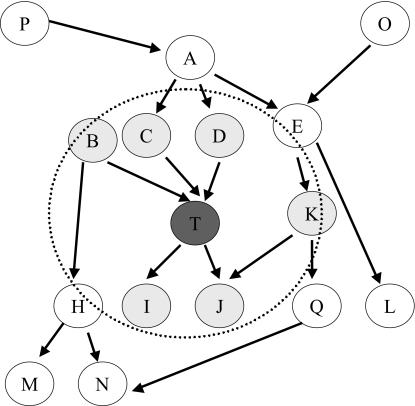
Markov Blanket of response variable T. All variables (except O) in the above graph are univariately associated to the response variable T (and thus predictive of T) and O is also multivariately predictive of T (in all models with at least one among {E, J, N, K, Q, L} as a covariate). Such situations can be very confusing to feature selection algorithms. However, the Markov Blanket of T, depicted as the gray-shaded nodes within the circle around T, is provably (under broad conditions) the minimal set of predictors required for optimal prediction of T. The Markov Blanket eliminates redundancies thoroughly and systematically and connects structural with predictive models in a formalized manner (see text).

Contrary to simply detecting over-fitting, feature selection methods, when appropriately applied, prevent it: the smaller the number of available predictors, the harder it is for most learners to over-fit, thus elimination of irrelevant (hence redundant) predictors in general reduces the risk for over-fitting. This is because all other things being equal, smaller number of predictors leads to classifiers with smaller complexity (eg, in order to construct a classifier from one predictor, one often has less modeling choices than for construction a classifier from 1,000 predictors). Starting from formalized or heuristic notions of relevancy, numerous algorithms have been invented to choose only the variables that are relevant to the predictive task in question (eg, predict response to treatment from mass spectrometry or gene expression microarray data) while discarding the irrelevant (or relevant but redundant) predictors. Algorithms that do so by examining the structure of the joint distribution of the data only are known *as filters*, algorithms that perform search and test subsets of predictors via application of a validation sample and a classifier of choice are known as *wrappers*, algorithms that perform predictor selection as part of fitting the classifier are known as *embedded* feature selection algorithms, while combinations also exist (see [Guyon, 2003] and [Tsamardinos, 2003a]). Out of numerous algorithms that have been devised and tested in mass-throughput data analysis we discuss here *Markov Blanket* induction algorithms as a particularly well-motivated theoretically and practically powerful family of methods for variable selection. *The Markov Blanket of a response variable is the smallest set of predictors which renders all other predictors (outside the Markov Blanket) independent of the response variable conditioned on the Markov Blanket.* The Markov Blanket of the response variable has been shown (under broad assumptions) to be the smallest set of predictors required for optimal response prediction (Figure 8). Theoretical details are provided in [Tsamardinos, 2003a]. The Markov Blanket not only solves provably the feature selection problem but also addresses in a principled manner the massive redundancy of predictors that characterizes bioinformatics mass-throughput data. Recent algorithmic developments in the field of Markov Blanket induction have produced algorithms that can identify the Markov Blanket correctly, in a sample and computationally tractable manner in mass-throughput datasets ([Aliferis, 2003a] and implemented in [Aliferis, 2003b], and [Statnikov, 2005b]).

These latest Markov Blanket algorithms are very robust to high-dimensionality. This is because they contain an elimination phase in which any predictor that is a candidate for Markov Blanket inclusion (candidates determined by strength of association with the response variable) is admitted to the Markov Blanket if it cannot be rendered statistically conditionally independent given any subset of the other candidates in an iterative process^6^. This is a powerful control mechanism against false positives due to multiple testing (ie, while many predictors will have univariate and multivariate associations with the response strictly due to chance, it is in practice extremely unlikely that these chance associations persist when we condition on multiple other subsets of predictors that likely belong to the Markov Blanket). This has been verified in experiments with both real and simulated datasets, see for example [Aliferis, 2003a], [Tsamardinos, 2003b], and [Tsamardinos, 2003c]. The Markov Blanket admits both linear and non-linear multivariate functions of the response given the selected predictors. Perhaps most importantly from a biological interpretability perspective, under broad distributional assumptions the Markov Blanket has a localized mechanistic interpretation (ie, it contains the measured local causes and effects as well as local causes of local effects of the response variable, see Figure 8 and [Tsamardinos, 2003a]).

We repeat for emphasis that a feature selection procedure (especially of the wrapper variety, see [Tsamardinos, 2003a]) itself can be over-fitted to the data (ie, predictors can be selected in such a manner that the selection conveys information about the test data thus contaminating the model fitting process and leading to over-fitting) (see [Simon, 2003] and [Ambroise, 2002]). *For this reason, feature selection (and as a general rule any data analysis preparation step that needs more than one sample instance at a time) must be accomplished in training data only.* This directly follows from our discussion of over-fitting by multiple validation.

Finally, we note that any modeling choice with a free parameter can be over-fitted. For example, selection of genes for a concise model can be thought of a specific parameterization of the model (see [Ambroise, 2002] and [Zhu, 2006]). These authors have popularized in the bioinformatics literature the term “selection bias” for the over-fitting of the gene selection parameter. In our discussion we use the term “over-fitting of gene selection” instead, in order to avoid possible confusion with the broader meaning of “selection bias” in the statistical literature where it denotes non-random sampling of the data from the population.

#### (d) Dimensionality reduction

**Figure 9.**
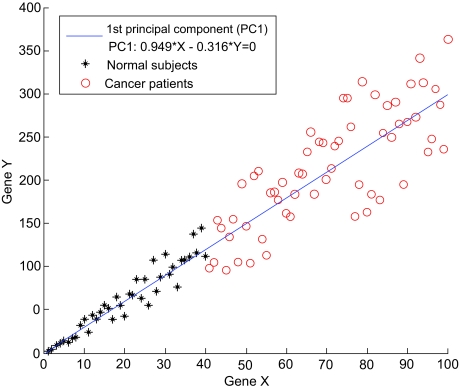
Reduction of dimensionality by Principal Component Analysis. We are given data for two genes X and Y for cancer patients and normal subjects. We are rotating the original axes to obtain new directions (principal components) corresponding to a more parsimonious description of data. The principal components lie along the directions of maximal covariance. The 1^st^ principal component (PC1) is shown on the figure and is defined as0.949*X-0.316*Y = 0. This principal component accounts for more than 90% of variance observed in the data. Thus one can replace values of the two genes X and Y by a single principal component. Notice that principal component analysis is not designed for feature selection since just one gene by itself is sufficient to discriminate cancer patients from normal subjects, however both genes receive nonzero weights in the 1st principal component.

This set of techniques reduces the number of predictors not by discarding some of them as in feature selection but by projecting all predictors to a new and typically more compact space that has some attractive properties. These properties typically are: (1) a few of the predictors (dimensions) in the new space can maintain essentially all dynamics observed in the full data; (2) the predictors in the new space can be independent from each other thus facilitating classical regression techniques by means of eliminating multicollinearity; (3) the number of predictors in the new space is constrained by the minimum of the number of samples and number of predictors in the original space thus allowing to restore over-specificity necessary for the regression modeling. The most established tools for dimensionality reduction are: Principal Component Analysis (abbreviated as PCA; also known as Singular Value Decomposition in linear algebra) [Shlens, 2003], Independent Component Analysis [Hyvärinen, 1999], and related methods. A simple example of use of PCA for the reduction of dimensionality is shown in Figure 9. We would like to emphasize that PC A is not designed to be used for feature selection. As it is seen from Figure 9, just one gene by itself is sufficient to perfectly discriminate cancer patients from normal subjects. However, both genes receive nonzero weights (also known as “loadings”) in the 1st principal component defined as 0.949*X–0.316*Y = 0, where X and Y denote genes X and Y, respectively (see Figure 9). Therefore, selection of genes that receive nonzero weights in a principal component will not in general provide a minimal-size set of optimally-predictive set of predictors. This is expected, in a way, since PCA is inherently an unsupervised technique not optimized for prediction of any one outcome, but rather tries to compactly represent the joint distribution of all variables. Furthermore, given that each principal component is a linear combination of predictors, it is difficult to interpret principal components biologically (ie, what does it mean that a gene appears with different loadings in several principal components, and how different loadings in different principal components compare across genes?). Another point is that while some authors have proposed that PCA is inherently resistant to over-fitting, in small samples principal components are themselves subject to estimation problems. This results in principal components that are inaccurate estimates of the true (ie, general population) ones, hence multiple validation search strategies for finding optimal principal component sets may need be applied to optimize classification performance opening the door to over-fitting [Nguyen 2002].

It is worthwhile to mention that some machine learning methods such as Neural networks can be configured to exploit dimensionality reduction implicitly (eg, by using fewer hidden units than input units in multi-layered feed-forward NN architectures) [Bishop, 1995].

Forward and backward model selection (which is a special form of “wrapper”-style feature selection) procedures have been criticized unfavorably to PCA by some authors on the basis of several accounts the most important of which is over-fitting the data (for example see the discussion in [Harrell, 2001]). Indeed if extensive model selection and fitting are performed at the same time (as often done by widely-used statistical software), the resulting model will be likely over-fitted in selected predictors parameter values and overall fit (or classification error) estimates. However, as long as the stepwise procedure is conducted separately from model fitting and error estimation the final model and its error estimates (respectively) will not be over-fitted [Reunanen, 2003].

#### (e) Penalizing complexity/parameters

One method for avoiding over-fitting without resorting to low-performance high-bias classifiers is to design and apply learners that trade-off complexity for “goodness-of-fit” of the data. Such learners are able to produce models that capture complicated functions of the data, however, they penalize for this extra complexity. Thus, everything else being equal, they exhibit a preference for simpler models and only generate a more complicated model if the additional complexity is justified by a substantial increase in explaining (fitting) the data. Notice that this practice is common in manual model selection in statistics. In Machine Learning however (and some statistical procedures that employ parameter shrinkage), it is embedded and formalized within the classifier. Examples of this practice is the Bayesian scoring for selecting a Bayesian Network to use for classification (or other Bayesian classifiers), the minimum description length principle applied to many different classifiers, the weight-decay strategy in training artificial neural networks, and others.

**Figure 10.**
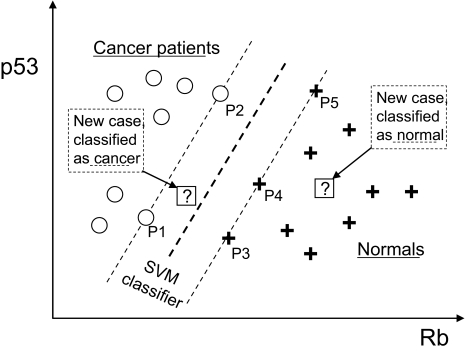
An example of a Support Vector Machine model. The model is denoted by the bold dashed line. The predictors are the expression levels of genes p53 and Rb. Any sample on the left of the line is classified as a cancer patient. Any sample on the right is classified as normal. The training samples are denoted by circles and crosses for cancerous and normals respectively. The model (discriminating line) produced by the SVM is the one that maximizes the margin of separation between the two classes. The samples on the margin (P1, P2, P3, P4 and P5) are called *support vectors.*

It is worth mentioning here kernel methods and specifically the case ofSupport Vector Machines [Vapnik, 1998] as prototypical examples of learners that penalize complexity. The term used for penalization of complexity in the literature for such methods is *regularization.* There are three main ideas in the Support Vector Machine classifier. The *first* is to use for discrimination a linear classifier (a line in two dimensions, a plane in three dimensions, and a hyperplane in general); specifically to use the hyperplane that maximizes the margin of separation between the classes. An example is shown in Figure 10. The *second* idea is to map the data to a much higher dimensional space called *feature space* before constructing the separating hyperplane, where they are linearly separable, ie, able to be separated by a hyperplane. The breakthrough achieved by kernel methods is that this mapping is done implicitly via a kernel function and thus it is extremely efficient: it is possible to classify new data using hyperplanes in feature spaces of billions of dimensions in times typically of less than a second. The *third* important idea in SVMs is to allow for misclassifications when the data are not linearly separable and trade-off misclassification for a wider margin of separation of the rest of the data (this is the “soft-margin” formulation of SVMs). Extensions of the standard SVM exist for multi-class and regression problems (eg, see [Platt, 2000], [Kressel, 1999], [Weston, 1999], [Crammer, 2000], and [Smola, 2004]).

An important technical detail of the SVMs is that the hyperplane produced is a linear combination of the training samples. A hyperplane can be described by its normal vector *w* and so the previous statement means that *w* = ∑α*_i_* φ(*x_i_*), where *a_i_* are the coefficients that produce the hyperplane that maximizes the margin, *x_i_* are the input training sample vectors, and φ(*x*_i_) are their images in feature space. Since, as we mentioned the feature space typically has billions of dimensions (eg, mass-throughput analysis where the original dimensionality is tens of thousands and the kernel may be a polynomial of degree 2 or 3) the weight vector *w* has billions of parameters. However, *the free parameters are actually the Lagrange multipliers that are as many as the training samples.* In other words, for each extra training sample an SVM gets, it allows itself an extra free parameter. In addition, it can be shown that the vector *w* is a linear combination of only the support vectors, ie, “boundary” data points that are relevant for definition of the SVM classifier, ie, the training samples lying exactly on the margin. Since this number is typically much less than the total number of training samples, the SVM (in a practical sense) also minimizes the number of free parameters that it tries to estimate. The above properties are corroborated by significant empirical evidence showing the success of SVMs in many data analysis tasks of mass-throughput and other data ([Statnikov, 2005a], [Joachims, 1999], and [Schölkopf, 1997]).

In contrast, classical statistical models do not only seek means to correctly classify the data but also an estimate of the process that generates our data (the so-called “generative model”). As a result in a classical regression framework we may not have enough data to estimate a model unless the number of training cases is at least an order of magnitude as large as the number of free parameters in our model (and the number of such parameters grows worst-case exponentially to the number of predictors, depending on how severe non-linearity the analyst is willing to admit in the model selection process).

#### (f) Label re-shuffling

**Figure 11.**
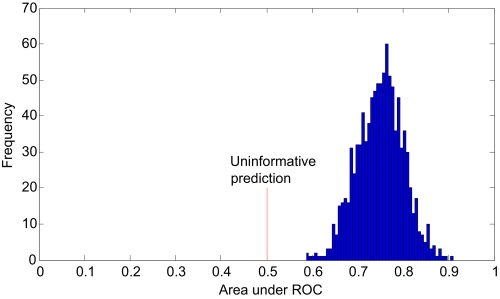
Results of label re-shuffling experiment indicative of over-fitting. The histogram describes the distribution of performance estimates using re-shuffled labels of the response variable for a hypothetical data analysis procedure. The mean performance is 0.75 area under ROC curve which is better than uninformative prediction (0.5 area under ROC curve).

Several permutation tests have been recently proposed to test significance of the achieved classification error (eg, see [Radmacher, 2002], [Mukherjee, 2003], and [Lyons-Weiler, 2005]). Here we are presenting a method not for significance testing of the achieved classification error, but for detecting and quantifying over-fitting. The method works as follows: Once a model is fit and its generalization error is estimated, the original response variable labels are randomly re-shuffled. The data analysis process is repeated a large number of times, each time with newly-permuted labels for the response variable. If the mean error estimate is better than uninformative (eg, 0.5 area under the ROC curve) more often than what expected by chance, then the data analysis process is over-fitted [Aliferis, 2003c]. The difference between the mean error estimate and uninformative classification error value can be used as a measure of over-fitting. See Figure 11 for an example.

### 3.3. Curse of Dimensionality

The term “Curse of Dimensionality” refers to the increased difficulty (or even, in the worst case, *collapse*) of statistical or machine learning methods due to very large number of predictors. In addition to the problem of large number of falsely positive associations and to over-fitting (both described in section 3.1) that are related to (and partially caused by) large predictor numbers, several additional problems are incurred as described below:

#### (a) Under-specified models

**Figure 12.**
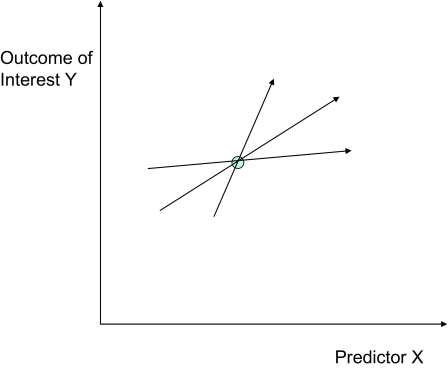
An example of an under-specified model. When there are two dimensions (the predictor X and the outcome Y) but only one training sample (denoted by the circle), an infinite number of minimum least-squares lines is possible.

Certain methods inherently require the number of samples to be larger than the number of dimensions. Otherwise, the model to be generated by the method is under-specified, ie, there is an infinite number of solutions, eg, a least-squares-based linear regression method in *d* dimensions with *k* < *d* available training samples. To make the example concrete let *d =* 2 and *k =* 1 as shown in Figure 12. Any line that crosses through the training example minimizes the least squares error of the regression. While additional constraints and preferences can be added to select a model, this is not trivial for all methods. Thus, many statistical modeling methods fail due to infeasibility of solving the linear system of equations (as a part of the model fitting stage).

#### (b) Exponential sample requirements

Some learning methods such as decision trees require sample that grows exponentially to the depth of the tree. Since the depth of the tree typically grows very fast to the number of available predictors, the more such predictors available, and especially if each predictor’s contribution to the final classification is relatively small (a situation that is not uncommon in bioinformatics datasets) then standard (greedy) decision tree learning algorithms (eg, ID3 and offspring) run out of sample before a tree with small error is fitted.

#### (c) Computational intractability to fully explore interaction effects/non-linearities

**Figure 13.**
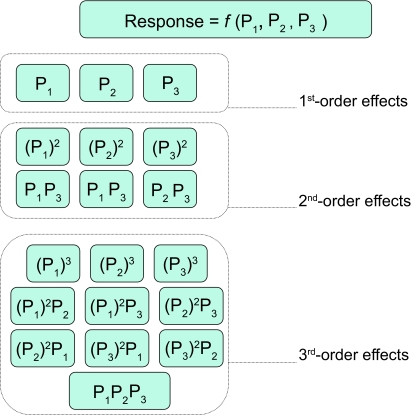
1^st^, 2^nd^ and 3^rd^ order effects of a non-linear predictor-response function with three predictors and up to 3^rd^ order effects (a “fully saturated” model that captures any non-linearity can be built from the set containing all the effects terms). Notice that the number of interaction terms grows faster than *2*^n^ and thus for any modeling task with more than two or three dozen variables, *exhaustive* estimation of the high-order effects cannot be accomplished in tractable computational time.

When considering interaction effects to capture non-linearities of the predictor-response functional form, as the predictors number increases, the number of n^th^ order interaction effects grows exponentially (assuming n is bounded only by the number of predictors and not by other considerations such as sample), rendering infeasible exhaustive examination of the interaction effects (see Figure 13). In predictor spaces with 10,000 predictors for example even 2^nd^ order effects become impractical to be examined exhaustively in tractable computational time [Agresti, 2002].

#### (d) Irrelevant of superfluous dimensions may severely affect methods that calculate distances in the input space

It is easy to see for example why the *k*-Nearest Neighbors as a learning method is sensitive to the number of dimensions of the problem. In order to classify a new case, *k*-Nearest Neighbors selects the *k* “closest” or “most similar” training examples to the new case. The case is then classified to the most common label among its *k* neighbors [Mitchell, 1997]. The distance between cases is typically measured by the Euclidean distance (or some other metric). Let us consider the task of predicting cancer in subjects using 1-nearest neighbor where the data contains the following predictors: smoking status, color of eyes, brand of car, and name of spouse. It is easy to see that when predicting a new case a neighboring training case that looks very similar across the (presumed irrelevant in our example) dimensions of color of eyes, brand of car, and name of spouse is the one that will determine the classification. *Similarity in the relevant dimension gets diluted by the presence of numerous irrelevant variables.*

By the same token, if there are many “equivalent” variables and multicollinarity, ie, correlations among predictors, similarity across these dimensions will weight heavier in determining classification while the other dimensions will be relatively ignored. Consider the case of the above example where the predictors are now smoking status (yes/no), yellow stains on fingers, daily costs of cigarettes smoked, and occupational exposure to carcinogens. The first three variables essentially convey the same information for the target but because they are repeated, similarity across these dimensions weights heavier than similarity across the last variable.

#### (e) Severely impeded heuristic search and optimization

Methods like Artificial Neural Networks and Genetic Algorithms that rely heavily on gradient-based (the former) or heuristic search (the latter) optimization of a data-fitting objective function, have higher chances of being trapped in a local optimum of the fitness space ([Mitchell, 1997], [Bishop, 1995]).

### 3.4. Methods to deal with the curse of dimensionality

A number of powerful counter-measures have been devised to the curse of dimensionality. Most of these methods tend to address all the adverse effects of the curse of dimensionality namely increased danger of over-fitting, increased sample requirements, and increased computational complexity.

#### (a) Feature selection

Feature selection was discussed in section 3.2(c) Apart from reducing the propensity to over-fit, feature selection also improves computational efficiency as well as reduces sample size requirements for analysis. It also enables easier biological interpretation and focusing subsequent experimentation on a more manageable set of features (eg, molecules) and biological hypotheses about their roles and interactions.

#### (b) Dimensionality reduction

Just like feature selection, dimensionality reduction (explained in section 3.2(d)) reduces the propensity to over-fit, improves computational efficiency, eliminates unwanted predictor multicollinearity, reduces sample size requirements and restores conditions for fitting classical regression models. However it does not lend itself to a convenient biological interpretation as explained in section 3.2(d). In addition, practical dimensionality reduction methods may not be fully immune to the curse of dimensionality. For example [Nguyen, 2002] shows empirical results suggesting that PCA is not fully insensitive to many irrelevant features in microarray analysis.

#### (c) Methods robust to high dimensionality

We showed in section 3.2(e) how kernel-based methods, the primary practical exemplar of which are Support Vector Machines (SVMs) control over-fitting by regularization and control of the complexity of the learned classifier to adjust for the available data. We also described in section 3.1(c) one of several bounds for SVMs that are independent of the dimensionality of the learning problem [Schölkopf, 1999]. To intuitively understand how this can be the case and how it is possible to learn good classification and regression models with many more predictors than samples with SVMs, recall that an SVM classifier is a decision surface that separates the data classes well. However the surface is not defined by estimating some population parameter estimated by the data samples (that would be a “generative model” – oriented classical statistical approach). Instead the surface is fully defined by a subset of the samples (represented geometrically as vectors of instantiated predictor variables, and denoted as “support vectors”). Because the complexity of the model is always bound by the available sample (since one cannot have more support vectors than the available samples) the model complexity is inherently controlled by the learning method’s structure and does not have to be retro-fitted by feature selection or dimensionality reduction schemes (such as PCA for classical regression models). In addition, SVMs possess additional attractive properties such that (i) the optimization of their objective function is not heuristic (as for example in ANNs [Duda, 2001] or GAs [Hart, 1991]) but guaranteed optimal via efficient quadratic optimization, (ii) they examine high-order interaction effects *not exhaustively but implicitly*, and thus tractably, by means of the “kernel trick” whereby the data is projected to a higher-dimensional space, an optimal decision surface is identified there, and the solution is projected back to the original predictor space [Vapnik, 1998]. Since the projection is achieved via kernel functions utilizing dot products of the data, *the operation is quadratic to the sample, and only linear to the dimensionality for an arbitrarily large interaction effect order.* Further, since the kernels can be organized in mutually subsuming classes of progressively increasing complexity, the analyst (typically using a small number of cross-validation or theoretical error estimates) can efficiently identify the smallest-complexity decision surface that is likely to yield optimal generalization error. Despite all these attractive properties, SVMs can still in some cases be sensitive to the curse of dimensionality (see [Hastie 2001] for an example) and can benefit by additional feature selection to their regularization (see [Hardin 2004], [Guyon, 2002], and [Rakotomamonjy, 2003]).

#### (d) Heuristic methods

Various techniques from the fields of Operations Research and Non-Linear Optimization have been applied to neural network optimization as alternatives to the standard gradient descent optimization (for example, see [Fletcher, 1964], [Powell, 1997], [Beale, 1972], [Moller, 1993], [MacCay, 1992], and [Foresee, 1997]). Also weight decay, momentum, random restarts and other heuristic and quasi-heuristic methods have been devised to augmentANN fitting with very large dimensionalities (see [Mitchell, 1997] and [Hagan, 1996]).

#### (e) Expert filtering based on domain knowledge

If in some area of research experts can guarantee that some predictors are irrelevant or very likely to be insignificant, then the data analyst can eliminate those. Unfortunately in molecular medicine, where very often so little is known about the processes studied, this entails the danger of missing potentially significant determinants of disease or outcome. However under special circumstances, for example in SNP arrays, SNPs belonging to a specific haplotype can be replaced safely by the “tagSNP” of that haplotype as long as the tagSNPs are identified with stringent statistical criteria [Gibbs, 2003].

#### (f) Newer algorithms to fit regression models

Several newer algorithms have been introduced to fit classical regression models using very high-dimensional data, eg, [Komarek, 2005], [Komarek, 2003], [Zhu, 2005], [Shevade, 2003], [Genkin, 2004], [Zhang, 2003]. The above methods are fairly insensitive to the curse of dimensionality.

### 3.5. Causality versus Predictiveness

While often purely predictive models are quite satisfactory for supporting decision making, (eg, biomarkers that are predictive of outcome are valuable since they can be used as surrogate endpoints in clinical trials) a significant part of the data analysis endeavor is geared toward discovery of mechanistic, ie, *causal* knowledge. Thus the importance of causal discovery from observational data such as typical case-control mass-throughput data cannot be understated.

In the biomedical and biostatistical communities it is widely accepted that the final judge of causal (as opposed to predictive or associational) knowledge is the *randomized controlled experiment.* Such experiments can help discover causal structure: for example, if smoking is enforced to a random group of subjects that later exhibits increased lung cancer rates relative to a comparable random group that does not smoke, then smoking must be causing cancer, and the association cannot be attributed solely to other factors, eg, a gene that causes propensity to smoking and susceptibility to lung cancer. However, quite often experimentation is impossible, impractical, and/or unethical. For example, it is unethical to force people to smoke and it is impractical in the context of our discussion to perform thousands of experiments manipulating single and combinations of genes at a time in order to discover which genes cause disease and how they interact in doing so.

For these reasons some researchers have been using a number of heuristic methods for causal discovery. Typically, heuristic methods used are based on *classification, concept formation,* and *regression* methods (rule learning [Mitchell, 1997] and [Holmes, 2000], clustering [Eisen, 1998], logistic regression [Agresti, 2002]). The resulting models are interpreted causally in a variety of subjective ways. From our survey of the literature, the four most prominent heuristics for causal discovery in biomedicine are:

- Heuristic 1 = “If they cluster together they have similar or related function.”
- Heuristic 2 = “If *A* is a robust and strong predictor of *T* then *A* is likely a cause of *T.*”
- Heuristic 3 = “The closer *A* and *T* are in a causal sense, the stronger their correlation.”
- Heuristic 4 = Surgeon’s General’s Epidemiological Criteria for Causality [U. S. Department of Health, Education, and Welfare, 1964]: “*A* is causing *B* with high likelihood if: (*i*) *A* precedes *B*; (ii) *A* is strongly associated with *B*; (iii) *A* is consistently associated with *B* in a variety of research studies, populations, and settings; (iv) *A* is the only available explanation for *B* (“coherence”); (v) *A* is specifically associated with *B* (but with few other factors).”

It can be shown that all these heuristic methods are not sound and may produce misleading results [Spirtes 2000]. Fortunately, it was shown, relatively recently (1980’s), that it is possible to soundly infer causal relations from *observational* data in many practical cases (see [Pearl, 1988], [Spirtes, 2000], and [Glymour, 1999]). Since then, algorithms that infer such causal relations have been developed that can greatly reduce the number of experiments required to discover the causal structure. Several empirical studies have verified their applicability: [Glymour, 1999], [Spirtes, 2000], [Tsamardinos, 2003 c], and [Aliferis, 1994]. In 2003 the Nobel Prize in Economics was awarded to C.W.J. Granger for his causal methods for time series analysis (first introduced in the late 60s). While the econometrics community has embraced computational causal discovery from observational data, research in the field is just beginning in biomedicine. In mass-throughput exploratory data analysis the need for causally informative methods is particularly pressing because the number of the available molecules is so large and their plausible interconnections so complex that a reductionistic single-molecule-by-experiment approach alone is woefully inefficient and expensive to address the overabundance of biological questions we can generate. For example, a set of 50 genes that are closely correlated with AML and ALL distinction were selected in [Golub, 1999]. However, the same authors acknowledge that both a smaller and larger set of genes can be used for AML/ALL classification as well. When it comes to biological validation of the obtained results, one usually has to focus on some specific subset of genes (ideally, of the small size). As discussed above, the causal discovery techniques provide this focused hint to the researchers.

In such datasets a number of formidable challenges still exist, such as how to scale-up existing algorithms for causal discovery from observation data to the number of variables encountered in biomedical domains, and the question to what degree the assumptions of the algorithms hold in these domains.

In general, formal methods define a proper language for representing, reasoning, and talking about causality. This language has so far been established well for Bayesian Networks (and variations), which are directed acyclic graphs annotated with probability tables and coupled with a requirement (the “Causal Markov Condition”) that relates causal graph structure to independencies among variables in the data distribution modeled by the Bayesian Network (for technical details see [Pearl, 2000]). Interpretation of causation in such graphs is intuitive: a variable in the graph causes another variable *directly* if and only if there is an edge from the former to the latter (provided the graph does not contain unnecessary edges). While several algorithms have appeared that *canprovably* discover the full network of causal relationships of variables (eg, regulatory gene networks) under certain assumptions and given only *observational* data, these methods do not theoretically (in the worst case) [Chickering, 1994] or practically [Friedman, 1999] scale-up to more than a few hundred variables.

Researchers in our group have recently developed new principled causal discovery methods that soundly and provably induce causal relationships (under certain assumptions) from observational data and that scale up to hundreds of thousands of variables (see [Aliferis, 2003a], [Tsamardinos, 2003c], and [Tsamardinos, 2006]). Scaling-up is possible if one focuses on learning *local* causal structure, eg, the *direct* causes and effects of a disease or a gene of interest. The local methods are able to discover parts of a large network of relations, which can be pieced together to provide a partial map of the full, *global* network. The advantages are scalability, and the ability to at least be able to learn the parts of the network for which this is practically feasible given the computational resources and available sample, and for which the assumptions hold.

In [Aliferis, 2003a], [Tsamardinos, 2003c], and [Tsamardinos, 2006] extensive experiments substantiating these statements are presented. In simulated experiments the causal structure that created the data is known and this makes it possible to compare the hypothesized causal structure output by the algorithms to the true structure. In these experiments, causal discovery methods have given very promising results.

Typical assumptions of causal discovery algorithms are (i) that all confounders of all variables connected directly to the response variable are observed (this property is called *local causal sufficiency*); if local causal sufficiency does not hold, then it is assumed that the information in the local neighborhood is enough to infer the presence or absence of specific confounding via existing algorithms (eg, the FCI algorithm [Spirtes, 2000] and the IC* algorithm [Pearl, 2000]); (ii) that dependences and independences in the data are completely captured by the causal Markov Property (a detailed explanation of what this principle entails is given in [Pearl, 2000]), and (iii) that there is enough sample relative to the functional forms among variables and the size of the causal neighbourhood (since the largest the latter is, the more sample is needed). While these assumptions are sufficient, there are not necessary and departures from them can be tolerated to various degrees depending on the nature of the data analysed ([Pearl, 1996]). It is also worthwhile to mention that one of the current challenges in application of causal inference algorithms to mass-throughput datasets is that statistical decision that are at core of these methods are sensitive to the measurement imprecision in the such datasets.

While it is certainly too early to conclude with certainty which existing or yet-to-be-discovered causal inference algorithms will prove to be most effective for mass-throughput data, it is safe to say that formal theories of causal inference, well-characterized algorithmic frameworks and resulting practical algorithms for computational causal discovery will play a significant role in deciphering mechanistic and structural aspects of mass-throughput data as opposed to strictly associational and predictive methods (for example, see a comprehensive bibliography on learning causal networks of gene interactions via formalized causal as well as heuristic regulatory induction algorithms [Markowetz, 2005]). As emerging examples of formal computational causal discovery tools, we cite the Causal Explorer toolkit (http://www.dsl-lab.org) or the Tetrad IV (http://www.phil.cmu.edu/projects/tetrad/).

### 3.6. Integrating heterogeneous data

Many researchers believe that the next generation of mass-throughput clinical molecular medicine models will rely on integrated analysis of the effects of clinical, demographic, microarray gene expression, SNP, proteomic, and other types of data on phenotypes of interest [Troyanskaya, 2005]. In particular, it makes sense to integrate distinct data sources when they provide additional information about the response variable. For example, when building models to predict disease outcomes, one should not solely rely on gene expression data, but can also include demographic and clinical data (eg, age of patient, gender, smoking status, stage of disease, medication data, etc.) and possible other data sources [Pittman, 2004]. The reason for doing this is the following: Apriori it is unknown what is the information content of different types of data with respect to the problem at hand. That is why the final model will be chosen among models that are constructed with different combinations of data types. For example, a study can evaluate and select the best among several models: Model 1 based on gene expression data, Model 2 based on gene expression and clinical data, and Model 3 based on gene expression, clinical, and imaging data, and so on.

**Figure 14.**
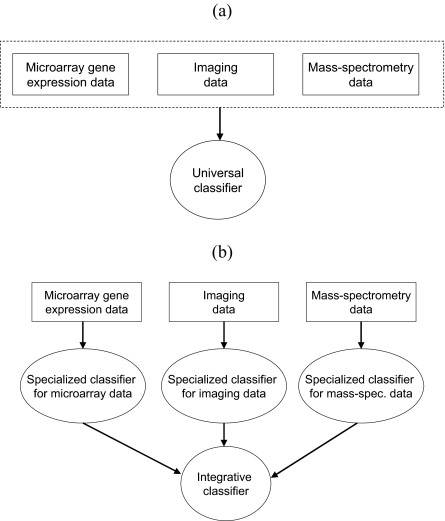
Two fundamentally different frameworks for integrated analysis of heterogeneous data: (a) simultaneous (tightly-integrated) analysis, and (b) sequential (loosely-integrated) analysis.

Usually, distinct data types are analyzed individually in the literature via quite dissimilar approaches. The bioinformatics literature in particular exhibits a high degree of method specialization to the idiosyncrasies of specific molecular assaying platforms (especially microarrays). There exist at least two fundamentally different frameworks for integrated analysis of heterogeneous data (Figure 14): (a) *tightly-integrated (simultaneous) analysis*, and (b) *loosely-integrated (sequential) analysis.* The former applies data modeling methods capable of handling the variety of data types while the later employs data-specific methods and then integrates the data-specific results. The advantages of tightly-integrated methods (wherever applicable) are that they lead to semantically coherent conclusions and, moreover, interactions of predictors belonging to separate assay/observational modalities (eg, interaction between expression abundance and SNPs) will not be missed. Loosely-integrated analyses can be attractive due to the existence of established and well-studied techniques for each type of dataset. However, they may miss interactions among predictors analyzed separately, they may lead to suboptimal prediction performance and may not control for data pre-processing (or other data-specific) incompatibilities between data types.

As one of several conceivable loosely-integrated frameworks we cite here using voting schemes to combine the results of data-specialized models since such schemes have been shown to perform well in general machine learning research. As one of several conceivable tightly-integrated frameworks we propose here the combination of SVMs with Markov Blanket induction algorithms. The rationale for this combination (in addition to the benefits related to preventing over-fitting and avoiding the curse of dimensionality conferred by feature selection and SVMs as explained earlier) is that under the condition of faithful distributions (which is the vast maj ority of distributions with very few known deviations known so far [Meek, 1995]), universal approximator learners (such as SVMs), and standard loss functions (ie, mean squared error, area under the ROC curve) the Markov Blanket predictors are guaranteed to be the smallest optimal predictor set and guaranteed to yield soundly local causal determinants as well [Tsamardinos, 2003a]. It does not matter what are the individual variables (clinical, demographic, gene expression, SNPs or other), as long as these assumptions hold, the predictors can be analyzed together. Furthermore, SVMs are general-purpose classifiers. While specialized kernels have been devised for analysis of specific data types (ie, special kernels for text or for sequence matching) valid combination kernels can be easily derived for the combined data by combining the data-specific sub-kernels [Briggs, 2005]. In practice simply rescaling the data linearly to the [0, 1] or [–1, 1] interval while breaking up nominal variables to “dummy” binary variables is often sufficient to achieve excellent results [Hsu, 2005]. Besides aforementioned theoretical benefits of using Markov Blanket and SVM methods for tightly-integrated analysis of heterogeneous data, we review the recent work of [Aliferis, 2003a] that proposed a new Markov Blanket method HITON and evaluated it across many types of biomedical data: biochemical (binding to thrombin), micro-array gene expression (lung cancer diagnosis), proteomics (prostate cancer diagnosis), clinical (arrhythmia diagnosis), and text. The authors found that (1) HITON reduces the number of variables in the prediction models by three orders of magnitude relative to the original variable set while improving or maintaining accuracy and (2) HITON outperforms the baseline algorithms by selecting more than two orders-of-magnitude smaller variable sets than the baselines, in the selected tasks and datasets.

### 3.7. Lack of standard protocols for data analysis

Finally, one of the major challenges in working with mass-throughput data is the absence of standard protocols for data analysis. Atypical analysis of such data involves a number of steps, for example mass spectrometry supervised data analysis may involve (but not limited to) the following steps: M/Z range restriction, baseline subtraction, normalization, peak detection, peak alignment, binning, feature selection, classifier construction, and classifier evaluation. However, researchers often have agreement neither on methods (and corresponding parameters) to be used in each step of data analysis nor on the specific sequence of steps (eg, see [Coombes, 2003], [Coombes, 2005], [Yasui, 2003a], [Yasui, 2003b], [Malyarenko, 2005], [Randolph, 2004], [Jeffries, 2005], [Wong, 2005], [Sauve, 2004], [Ressom, 2005], and [Hastings, 2002]). For example, while building classification models from mass spectrometry data some studies apply Decision Trees [Qu, 2002], others apply Support Vector Machines [Wagner, 2003]; some studies perform baseline subtraction and peak detection as a single integrated step ([Coombes, 2003] and [Koomen, 2005]), others perform these operations sequentially [Coombes, 2005], yet others do not perform baseline subtraction at all [Yu, 2005].

The lack of standard protocols for mass-throughput data analysis is also reflected in the available software (both academic and commercial). In a recent review of software systems for supervised microarray data analysis [Statnikov, 2005b], it was concluded that almost every software offers different methods and no system enforces unbiased error estimation while optimizing classification models and selecting relevant genes. This increases the risk of over-fitting and may lead to non-reproducible models and error estimates. Two attempts to circumvent these problems are GEMS [Statnikov, 2005b] and FAST-AIMS [Fananapazir, 2005] that we discuss below.

While considerable efforts have been made to develop data storage and dissemination standards (eg, the MIAME standard for microarray gene expression data [Brazma, 2001] and the Human Proteome Organization’s PSI for mass spectrometry data [Orchard, 2004], [Hermjakob, 2004]) there is limited agreement as to which are the best methods to process, analyze, and interpret data and which are high-performance protocols for putting together component techniques for a unified analysis. We believe that development and availability of standard protocols will facilitate high-quality analysis of high-throughput data similar to other areas of biomedical research (eg, clinical trials [Piantadosi, 1997]). We specifically propose that robust high-quality protocols for data analysis can be developed in a series of three consecutive and independent steps:

- Step 1 involves thorough evaluation of component algorithms, model selection schemes and error estimation procedures across many representative datasets for a specific problem area (eg, diagnosis of cancer from microarray or mass spectrometry data). For an example of such algorithmic evaluation, refer to [Statnikov, 2005a].
- Step 2 involves implementation and validation of the protocols in independent datasets and comparison to published analyses and new ones, including cross-dataset experiments. For an example of such an evaluation, refer to [Statnikov, 2005b] (note that this protocol is fully automated in the GEMS system in that study), [Semmes, 2005], [Paik, 2004], and [Li, 2005].
- Because standard protocols can ideally be automated to produce powerful software to help the biomedical researchers, Step 3 involves usability testing of the resulting software. For an example of such an evaluation, refer to a preliminary usability evaluation of the FAST-AIMS system for mass spectrometry analysis by [Fananapazir, 2005].

We emphasize that the above steps 1–3 should be iterated periodically to include new developments in the field.

Also notice that the process developed and evaluated above is by design independent of the problem context or specifics of the assay and can, in principle, be applied to various types of data. Promising results for this process have been obtained in the domains of microarray gene expression [Statnikov, 2005b] and mass-spectrometry [Fananapazir, 2005].

## 4. Conclusions

This paper is predicated on the thesis that sound, scaleable and reproducible data analysis of “-omics” mass-throughput data is essential for delivering the promise of molecular medicine both for cancer research and beyond. We have identified a handful of recurrent core challenges facing every research group entrusted with the analysis of such data. These include error estimation and over-fitting, the curse of dimensionality, the fundamental distinction of causal versus predictive modeling, the integration of heterogeneous types of data, and the lack of standard protocols for data analysis.

In an effort to shed light on the problems and indicate possible solutions we explained the basic factors leading to these problems and outlined established and emerging theoretical frameworks, focused methods and practical tools to address them.

In summary our main points can be stated as follows:

- All current error estimation procedures are sensitive to extremely small samples.
- Given a fixed sample size, avoiding over-fitting relies on techniques that control the complexity of the model and match it well to the available sample and complexity of modeling task.
- As a general rule, to avoid over-fitting, any data analysis preparation step that needs more than one sample instance at a time must be accomplished in training data only. For example, it is not advisable to perform feature selection with all (ie, training plus test) data.
- Contrary to wide-spread belief, when sample size is very small, “independent dataset validation” is not a thorough solution to the over-fitting problem: it neither prevents over-fitting nor always detects it.
- For all the above reasons, having sufficiently large sample is a critical factor in designing mass-throughput studies.
- Machine learning departs from classical statistics in mass-throughput data analysis and offers robust and computationally scaleable solutions often not currently feasible via traditional statistical modeling. Data analysts working with mass-throughput data should be aware of the relative strengths and weaknesses of statistical machine learning versus classical statistical techniques in order to produce the best possible analyses. Drawing from our experience, we recommend a collaborative team of biostatisticians and machine learning experts working together on challenging data analysis tasks not only to provide alternative models on the same data but also to combine the two. For example, these collaborative teams can provide unbiased error estimates from statistical theory for powerful machine learning methods, or allow to base decisions of the machine learning techniques on the appropriate statistical tests.
- Another aspect of data interpretation, that of separating mechanistic (causal) from predictive modeling is necessary but quite undeveloped currently. We discussed recent algorithmic work in computational causal discovery that has great promise for such analyses.
- In the near future many different types of clinical, molecular and imaging data will have to be analyzed in an integrated fashion. We described two fundamentally distinct approaches with different strengths and weaknesses: the tightly-integrated and the loosely-integrated frameworks. We suggested that the Markov Blanket feature selection & SVM classifier framework is one promising approach for tightly integrated analysis, while voting schemes a promising approach for loosely-integrated analysis.
- Finally we emphasized the need for standardized analysis protocols and benchmarks. We discussed a three-step process for the development, validation and automation of such protocols and gave early examples from the literature that employed it as well as evaluated its results by validating the produced models in independent datasets, a gold standard that applies to all scientific claims.

As the collective knowledge in the field improves, the problems we identified will surely become less salient but always important to address because of their fundamental nature. We believe thus that no successful analysis can overlook these fundamental aspects of making sense of mass-throughput datasets. Existing and new yet-to-be-developed methods will certainly succeed or fail in large part to the extent by which we address these challenges successfully.

## Appendix I: Glossary & Abbreviations

- **Accuracy** – a very commonly used performance metric. It is defined as the number of correctly classified samples divided by the total number of classified samples. Sometimes “accuracy” is used as a synonym for “performance.”
- **ANN** – Artificial Neural Networks.
- **AUC** – Area under ROC curve.
- **Causal mechanism** – a special type of relationship connecting two or more variables. Informally, “*A causes B*” is an example such mechanism and means that manipulations (ie, forcing or guaranteeing a specific value for) *A* will have a predictable effect on (ie, will constrain in a well-defined way) the distribution of *B.* Note that the specific manner in which the distribution of *B* changes is semantically (ie, functionally or biologically) unconstrained.
- **Classification (or regression) error** – error produced by a classification (or regression) model in prediction of the outcome variable in a set of samples. This error indicates how well the model classifies discrete outcome variable (classification) or how well the model approximates a continuous outcome variable (regression).
- **Classification learning algorithm** – an algorithm that creates models from data capable of predicting a discrete outcome variable as a function of predictor variables.
- **Dimensionality (of a dataset)** – the number of variables in the dataset.
- **Empirical error** – the error obtained in training data.
- **Feature (or variable)** – a coded descriptor of a quantitative or qualitative characteristic recorded for each observational unit of interest. For example: age, cancer status, expression value of BRCA1 gene, recorded for each patient in a cohort.
- **GA** – Genetic Algorithm.
- **Generalization error** – the error in the population where all data comes from.
- **Generative model** – a model that captures conditional probability of class given input patterns. Alternatively, a model that captures a joint probability distribution of the input and class variables.
- **Heuristic** – a rule of thumb or method that does not guarantee optimal solutions to a problem all of the time, but passable solutions some of the time.
- **Hyperplane** – a sub space V of a vector space W, such that V has one less dimension than W. In 2D space a hyperplane is a line; in 3D space a hyperplane is a plane, etc. A hyperplane can also be thought as a surface that separates a geometrical space in two parts. The hyperplane can be used as a decision surface that indicates in which part of the space data points with a particular value for the response variable belong to.
- **KNN** – K-Nearest Neighbors.
- **Learner performance (or error metric)** – a function that expresses how well the learner performs. Examples are: accuracy, area under the ROC curve, mean squared error, relative classifier information, positive and negative predictive values, etc.
- **Linearly separable data** – set of data samples that can be separated (according to categories of the outcome variable) by a hyperplane. The hyperplane is defined in the space with dimensions corresponding to the predictor variables.
- **Multivariate association** – association between two sets of variables (such that at least one set has 2 or more variables).
- **Observational data** – data that is not produced by experiments but occur naturally and are simply observed and recorded. A case-control dataset with cancer patients and normal controls chosen from a hospital population is an example of observational dataset.
- **Outcome (or response, or target, or dependent) variable** – A variable that researchers are interested in predicting as a function of predictor variables.
- **Predictor variable (or predictor, or independent variable)** – A variable used for prediction/modeling of an outcome (ie, response) variable, alone or in combination with other predictors.
- **Randomized controlled experiments** – a special kind of experiment in which the experimenter attempts to establish causal mechanism knowledge of the form “*A causes B*” by forcing *A* to take various values from its possible value range randomly and then observing whether *B* differs among groups that have different values for *A.* Typically observational units with values of variable *A* that denote absence of *A* are considered “controls” for units with values of variable *A* denoting presence of *A.*
- **Regression learning algorithm** – an algorithm that creates models from data capable of predicting a continuous outcome variable as a function of predictor variables.
- **ROC curve** – Receiver Operating Characteristic curve.
- **Sample (or data point, or instance, or data vector)** – a vector containing values for all measured features. Also: a set of data points sampled from (ie, either chosen randomly or selected non-randomly) the general population (ie, the set of all possible data points). Which of the two meanings is intended can be inferred from the context.
- **SVM** – Support Vector Machines.
- **Testing set/data/dataset/sample** – portion of the data (subset of samples) used to estimate how well a previously trained and validated model will perform in future independent samples from the same population.
- **Training set/data/dataset/sample** – portion of the data (subset of samples) used to develop (ie, “train” or fit parameter values for) a classification or regression algorithm.
- **Univariate association** – association between a pair of variables.
- **Validation set/data/dataset/sample** – portion of the data (subset of samples) used to estimate how well a previously trained model fits the data.

## Footnotes

1 An indicative search performed on 11-2-2005 in PubMed yielded 14,932 articles mentioning “Oligonucleotide Array Sequence Analysis” as a MeSH term. Out of those articles 1,105 references were made to statistical machine learning methods and specific classifiers such as SVMs, Neural Networks, Clustering, etc. while 353 references were made to the predominant classical statistical methods of Linear regression, ANOVA and Discriminant function analysis.

2 In the remainder of this paper we deal with statistical machine learning concepts exclusively.

3 A widely-recognized form of over-fitting due to multiple validation is false univariate associations of a variable of interest (eg, a gene expression value) with the response variable (eg, survival) due to multiple statistical comparisons. It is a special sub-case of over-fitting in the sense that any valid (ie, non-random and thus, given enough sample, statistically significant) univariate association corresponds to a univariate predictive model for the response variable that should generalize to fresh samples from the same population of patients. False discovery rate (FDR) techniques such as ones introduced in [Storey, 2003] and variants can and have been used to address the problem for the univariate case as and thus will not be repeated here. We will focus instead on the issue of over-fitted multivariate models.

4 This has also called ‘overtuning’ when multiple predictive models are evaluated on the same test set.

5 In gene expression and many high-throughput datasets, every patient has a unique profile. Thus, its bootstrap sample contains multiple instances of the same patients, which is not representative of the general population. This leads to a bias that is not currently known how to correct for.

6 This description is by necessity somewhat over-simplified. For technical details please see [Aliferis, 2003a], and [Tsamardinos, 2003c].
